# Comorbidities and the risk of death among individuals infected by COVID-19 in Espírito Santo, Brazil

**DOI:** 10.1590/0037-8682-0138-2021

**Published:** 2021-07-02

**Authors:** Edson Zangiacomi Martinez, Davi Casale Aragon, Carolina Moreira Pontes, Altacílio Aparecido Nunes, Ethel Leonor Noia Maciel, Pablo Jabor, Eliana Zandonade

**Affiliations:** 1 Universidade de São Paulo, Faculdade de Medicina de Ribeirão Preto, Ribeirão Preto, SP, Brasil.; 2 Universidade Federal do Espírito Santo, Departamento de Estatística, Vitória, ES, Brasil.; 3 Universidade Federal do Espírito Santo, Programa de Pós-Graduação em Saúde Coletiva, Vitória, ES, Brasil.; 4 Instituto Jones dos Santos Neves, Vitória, ES, Brasil.

**Keywords:** COVID-19, Coronavirus disease, Comorbidities, Chronic diseases, Epidemiology, Survival analysis

## Abstract

**INTRODUCTION:**

We investigated the association of self-reported comorbidities with fatality risk among individuals infected with Coronavirus disease 2019 (COVID-19) in Espírito Santo State, Brazil.

**METHODS:**

We included 212,620 individuals, ≥30 years old. The data were obtained from the COVID-19 panel. Kaplan-Meier curves and Cox regression model were used.

**RESULTS:**

COVID-19-positive individuals presenting with chronic conditions were at a higher risk of fatality than individuals without these comorbidities. Age had a significant effect on these relationships.

**CONCLUSIONS:**

Comorbidities were associated with an increased risk of fatality. Middle-aged people (30-59 years) with comorbidities should also be considered as a vulnerable group.

Coronavirus disease 2019 (COVID-19) first appeared in China in December 2019 and then spread worldwide. The pandemic arrived in Brazil at the end of February 2020, and 1 year later, the country had a cumulative total of 10,195,160 cases and 247,143 deaths from the disease. COVID-19 can lead to significant mortality in critically ill patients, especially those with comorbidities, such as hypertension and other cardiovascular diseases, diabetes, and obesity^1^ ^,^ ^2^. The rising prevalence of obesity in many countries^3^ has led to an increase in other chronic diseases, and studies regarding the possible influence of these morbidities on the incidence and mortality due to COVID-19 can provide a better understanding of the disease, and therefore, help develop strategies for its mitigation.

This study aimed to describe the association between self-reported diabetes; obesity; smoking; and pulmonary, cardiac, and renal comorbidities; and the risk of death among adults ≥ 30 years of age, and confirmed COVID-19 patients living in the state of Espírito Santo (ES), Southeast Brazil. ES is a coastal Brazilian state with an area similar to that of Switzerland (46,095 km^2^) and an estimated population of 4 million inhabitants in 2020. We used data from the COVID-19 Panel^4^ from the state government of ES. We considered individuals with a diagnostic date from February 22, 2020, to February 18, 2021, and a follow-up duration of up to 60 days. This study was conducted with anonymized secondary data and, therefore, did not require approval from the Human Research Ethics Committee.

The original dataset obtained from the COVID-19 panel had 988,818 records. After excluding the non-confirmed cases of the disease, those <30 years old, and inconsistent or missing data, we obtained a final sample of 287,836 individuals. Because 81 deaths were reported among 75,096 people < 30 years, a relatively small number, we decided not to include this age group in the present study. The cases of the disease were confirmed by real-time PCR, immunological methods, or antigen research. Individuals for whom laboratory confirmation could not be performed were classified as positive if they had influenza syndrome and a history of close or household contact 14 days prior to the onset of signs and symptoms with a person who was laboratory-confirmed for COVID-19. More details can be found in a technical note published by the Health Surveillance Management of the state government of ES^5^. Survival curves were estimated using the Kaplan-Meier (KM) method^6^, considering the time in days from diagnosis to death as the outcome of interest. These curves were used to estimate the proportion of survivors at each time period, where deaths due to causes other than COVID-19 or disease recovery were considered as censored data. The Cox proportional hazard regression model was used to identify variables influencing the proportion of survivors^6^. The results were expressed as hazard ratios (HRs) with 95% confidence intervals (95%CI). We checked the assumption of hazard proportionality by visualizing plots of log{-log[S(t)]} against log(t), where S(t) is the KM survivor function. Parallel lines for each class of a given variable suggest proportional hazards. R software was used to clean and organize the dataset and for statistical analyses.

Table 1 shows the proportions of individuals who self-reported comorbidities (number of cases per 100 individuals infected by COVID-19), with 95% CIs calculated using the exact binomial method. The proportion of individuals reporting diabetes was higher among older people (≥ 60 years), with no major differences between men and women. The proportion of individuals reporting renal, pulmonary, and cardiac comorbidities was similar among men and women, and the results showed an increase in the proportions across age groups (Table 1).

**TABLE 1:** Proportion of self-reported comorbidities among the studied population (*n* = 287,836).

	Age groups	Males			Females		
Comorbidity	(years)	n ^a^	Pr ^b^	95%CI	n ^a^	Pr ^b^	95%CI
							
**Diabetes**	30 - 39	32,642	1.00	(0.90 - 1.12)	38,018	1.60	(1.48 - 1.43)
	40 - 49	26,168	3.77	(3.54 - 4.01)	31,177	4.42	(4.19 - 4.65)
	50 - 59	18,532	9.42	(9.00 - 9.85)	22,748	10.63	(10.23 - 11.03)
	60 - 69	11,633	16.52	(15.85 - 17.21)	13,480	18.00	(17.36 - 18.66)
	70 - 80	5,369	20.08	(19.01 - 21.18)	5,908	23.32	(22.25 - 24.42)
	80 - 90	2,159	18.62	(17.00 - 20.33)	2,761	21.95	(20.42 - 23.54)
	>90 yr old	444	14.86	(11.69 - 18.52)	745	18.79	(16.05 - 21.79)
							
**Obesity**	30 - 39	32,631	2.28	(2.12 - 2.45)	37,986	3.08	(2.91 - 3.26)
	40 - 49	26,149	2.92	(2.72 - 3.13)	31,149	3.49	(3.29 - 3.70)
	50 - 59	18,523	2.84	(2.61 - 3.09)	22,735	3.66	(3.41 - 3.91)
	60 - 69	11,626	2.82	(2.53 - 3.14)	13,476	4.01	(3.69 - 4.36)
	70 - 80	5,363	2.59	(2.18 - 3.05)	5,900	4.39	(3.88 - 4.94)
	80 - 90	2,162	2.22	(1.64 - 2.93)	2,764	4.52	(3.78 - 5.36)
	>90 yr old	444	1.13	(0.37 - 2.61)	745	3.09	(1.97 - 4.60)
							
**Smoking**	30 - 39	32,646	1.64	(1.50 - 1.78)	38,016	0.95	(0.86 - 1.06)
	40 - 49	26,170	1.67	(1.52 - 1.84)	31,176	1.17	(1.05 - 1.30)
	50 - 59	18,532	2.08	(1.88 - 2.29)	22,748	1.58	(1.42 - 1.75)
	60 - 69	11,635	2.94	(2.64 - 3.26)	13,479	1.65	(1.44 - 1.88)
	70 - 80	5,366	3.78	(3.29 - 4.33)	5,910	1.42	(1.14 - 1.76)
	80 - 90	2,163	4.85	(3.99 - 5.85)	2,766	1.55	(1.13 - 2.09)
	>90 yr old	443	4.97	(3.14 - 7.42)	747	2.81	(1.75 - 4.27)
							
**Kidney comorbidity**	30 - 39	32,647	0.18	(0.14 - 0.24)	38,018	0.22	(0.18 - 0.27)
	40 - 49	26,172	0.39	(0.32 - 0.48)	31,179	0.38	(0.32 - 0.46)
	50 - 59	18,536	0.69	(0.58 - 0.82)	22,748	0.51	(0.42 - 0.61)
	60 - 69	11,636	1.46	(1.25 - 1.70)	13,482	0.88	(0.72 - 1.05)
	70 - 80	5,368	2.48	(2.08 - 2.93)	5,911	1.62	(1.32 - 1.98)
	80 - 90	2,161	3.79	(3.03 - 4.69)	2,766	2.46	(1.91 - 3.11)
	>90 yr old	444	4.28	(2.60 - 6.60)	747	2.14	(1.23 - 3.46)
							
**Pulmonary comorbidity**	30 - 39	32,639	1.26	(1.14 - 1.38)	38,018	2.49	(2.34 - 2.66)
	40 - 49	26,170	1.22	(1.09 - 1.36)	31,177	2.63	(2.46 - 2.81)
	50 - 59	18,529	1.49	(1.32 - 1.67)	22,744	2.87	(2.66 - 3.10)
	60 - 69	11,638	2.55	(2.27 - 2.85)	13,479	3.19	(2.90 - 3.50)
	70 - 80	5,366	4.12	(3.60 - 4.69)	5,910	4.59	(4.07 - 5.15)
	80 - 90	2,162	7.86	(6.76 - 9.08)	2,764	7.16	(6.23 - 8.19)
	>90 yr old	443	11.29	(8.49 - 14.61)	746	8.31	(6.43 - 10.53)
							
**Cardiac comorbidity**	30 - 39	32,641	4.86	(4.63 - 5.10)	38,019	5.75	(5.52 - 5.99)
	40 - 49	26,167	11.69	(11.30 - 12.09)	31,181	15.43	(15.03 - 15.84)
	50 - 59	18,531	24.04	(23.43 - 24.66)	22,747	29.04	(28.45 - 29.63)
	60 - 69	11,639	37.90	(37.02 - 38.79)	13,481	41.87	(41.03 - 42.70)
	70 - 80	5,369	48.24	(46.90 - 49.59)	5,910	52.05	(50.76 - 53.33)
	80 - 90	2,162	52.91	(50.78 - 55.04)	2,765	54.97	(53.10 - 56.84)
	>90 yr old	444	54.95	(50.19 - 59.65)	745	52.21	(48.56 - 55.85)

**Pr:** proportions; **95%CI:** 95% confidence intervals for PR; **yr:** years. ^a^ The total number of individuals in each group. Slight differences are due to missing values.^b^ Number of cases per 100 individuals.

Considering all individuals, the risk of death was higher among men (HR 1.48; 95%CI, 1.401.56). Sharma et al.^7^ reported that men infected with COVID-19 tend to have more severe diseases than women and a higher risk of death. According to these authors, these differences can be partly explained by the relatively higher contribution of pre-existing diseases among men, higher risk behaviors, occupational exposure, and inequities in the search for preventive care practices. Some authors have also studied the relationship between biological factors and the higher risk of death from COVID-19 in men^8^, but further studies are required to better elucidate these mechanisms.

Figure 1 shows KM survival curves for death stratified by educational level, age groups, and number of self-reported comorbidities (diabetes; obesity; smoking; and renal, pulmonary, and cardiac comorbidities). These plots are presented separately for men and women and include HR estimates with the corresponding 95%CI. Panels (a) and (b) of Figure 1 show that for both sexes, the risk of death increases with age. The KM cumulative fatality rates at 60 days after diagnosis were 1.1%, 5.5%, 14.9%, 41.7%, 79.0%, 74.3%, and 73.3% among men aged 30-39, 40-49, 50-59, 60-69, 70-79, 80-89, and ≥90 years, respectively. Among females, these rates were 1.4%, 2.7%, 9.5%, 26.2%, 47.4%, 63.1%, and 76.7%, respectively. Panel (c) of Figure 1 shows that illiterate women have a risk of death from COVID, which is equivalent to approximately 30 times the risk in literate women. Among males, the corresponding HR was estimated to be approximately 11.2. The KM fatality rates at 60 days after diagnosis were 52.2%, 41.5%, 37.5%, 17.4%, and 8.6% among illiterate males, and males with elementary school I, elementary school II, intermediate school, and higher education levels. Among females, these rates were 50.4%, 33.1%, 24.3%, 8.1%, and 4.3%, respectively. These findings highlight a deep association between the socioeconomic status of individuals and the risk of death from COVID-19. Panels (e) and (f) of Figure 1 show that the risk of death increases with the number of self-reported comorbidities, for both sexes.

**FIGURE 1:**
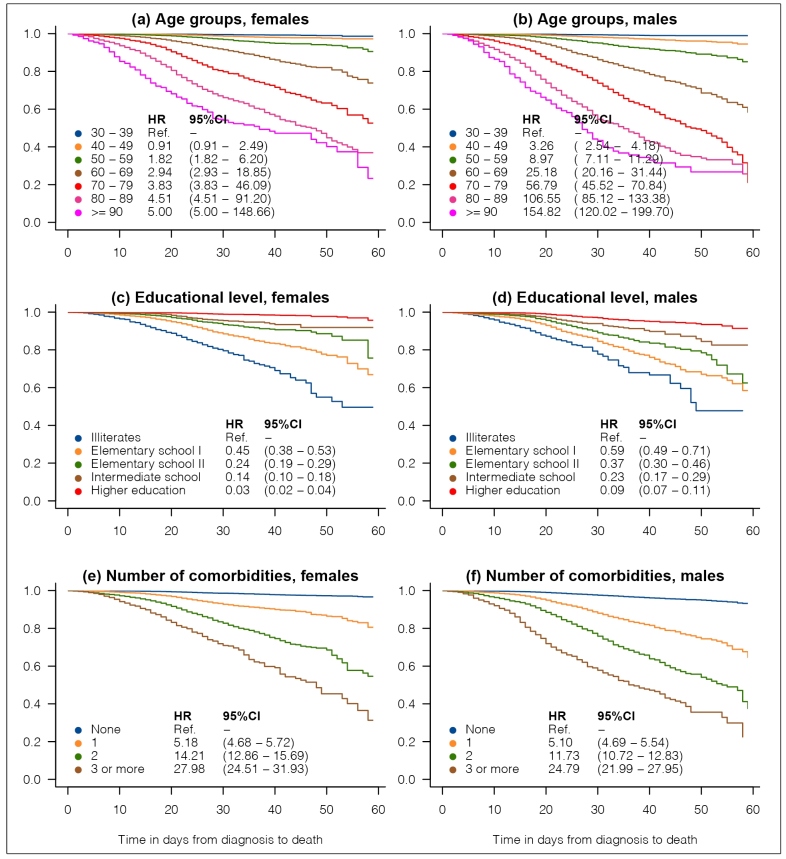
Kaplan-Meier curves for time to death. The graphs include estimates of hazard ratio (HR) with their respective 95% confidence intervals (95%CI) obtained from Cox proportional hazard regression models.

Table 2 shows estimates of HR for deaths due to COVID-19 comparing between individuals with and without each comorbidity of interest (diabetes; obesity; pulmonary, cardiac, and renal comorbidities; and smoking). Assumptions of proportional hazards were met in all model fits. Results presented in Table 2 reveal that comorbidities are closely associated with fatality in COVID‐19 patients. Overall, the results suggest that the middle-aged population (30-59 years) with comorbidities has an increased risk of death and should also be considered as a vulnerable group. However, HR values for the elderly also suggest expressive fatality risks. For example, individuals who are ≥90 years with renal comorbidities have a fatality risk that is twice that of those without these comorbidities (Table 2). 

**TABLE 2:** Estimates of HR for deaths due to COVID-19 comparing between individuals with and without each comorbidity of interest (*n* = 287,836).

	Age groups	Males		Females	
Comorbidity	(years)	HR	95%CI ^a^	HR	95%CI ^a^
					
Diabetes	30 - 39	16.23	(8.991 - 29.310)*	10.71	(6.033 - 19.020)*
	40 - 49	5.861	(4.228 - 8.125)*	7.451	(5.329 - 10.420)*
	50 - 59	3.609	(2.934 - 4.439)*	3.766	(2.972 - 4.771)*
	60 - 69	2.546	(2.206 - 2.939)*	2.974	(2.513 - 3.519)*
	70 - 80	1.695	(1.475 - 1.949)*	2.344	(2.001 - 2.747)*
	80 - 90	1.498	(1.257 - 1.785)*	1.757	(1.485 - 2.079)*
	>90 yr old	1.409	(0.983 - 2.010)	1.327	(0.990 - 1.779)
					
Obesity	30 - 39	10.35	(6.087 - 17.600)*	11.67	(7.221 - 18.800)*
	40 - 49	7.636	(5.461 - 10.680)*	7.784	(5.432 - 11.150)*
	50 - 59	3.380	(2.433 - 4.695)*	4.793	(3.541 - 6.487)*
	60 - 69	3.012	(2.346 - 3.867)*	3.517	(2.771 - 4.462)*
	70 - 80	2.373	(1.784 - 3.156)*	2.724	(2.120 - 3.499)*
	80 - 90	1.893	(1.279 - 2.802)*	1.981	(1.494 - 2.626)*
	>90 yr old	4.150	(1.697 - 10.150)*	1.172	(0.639 - 2.150)
					
Smoking	30 - 39	5.835	(2.692 - 12.650)*	4.062	(1.283 - 12.860)*
	40 - 49	5.685	(3.512 - 9.204)*	2.940	(1.302 - 6.641)*
	50 - 59	3.774	(2.660 - 5.356)*	3.522	(2.129 - 5.825)*
	60 - 69	2.826	(2.197 - 3.634)*	2.229	(1.426 - 3.483)*
	70 - 80	2.253	(1.796 - 2.827)*	2.219	(1.421 - 3.465)*
	80 - 90	1.924	(1.456 - 2.541)*	2.183	(1.412 - 3.375)*
	>90 yr old	1.755	(1.035 - 2.974)*	2.137	(1.221 - 3.738)*
					
Kidney comorbidity	30 - 39	30.41	(12.31 - 75.08)*	52.07	(26.07 - 104.00)*
	40 - 49	10.55	(5.893 - 18.90)*	12.14	(6.404 - 23.00)*
	50 - 59	6.910	(4.500 - 10.610)*	9.142	(5.526 - 15.130)*
	60 - 69	3.776	(2.821 - 5.055)*	5.250	(3.515 - 7.840)*
	70 - 80	2.537	(1.960 - 3.285)*	4.138	(3.035 - 5.641)*
	80 - 90	2.061	(1.536 - 2.765)*	2.743	(1.974 - 3.810)*
	>90 yr old	2.280	(1.295 - 4.013)*	2.043	(1.116 - 3.741)*
					
Pulmonary comorbidity	30 - 39	4.920	(1.993 - 12.15)*	1.796	(0.657 - 4.905)
	40 - 49	2.466	(1.162 - 5.235)*	2.808	(1.596 - 4.943)*
	50 - 59	4.012	(2.736 - 5.883)*	2.937	(1.952 - 4.420)*
	60 - 69	2.577	(1.980 - 3.353)*	1.562	(1.075 - 2.269)*
	70 - 80	2.144	(1.713 - 2.684)*	2.335	(1.813 - 3.009)*
	80 - 90	1.651	(1.302 - 2.094)*	1.553	(1.215 - 1.983)*
	>90 yr old	1.588	(1.075 - 2.346)*	1.550	(1.046 - 2.297)*
					
Cardiac comorbidity	30 - 39	4.802	(2.822 - 8.170)*	8.312	(5.301 - 13.04)*
	40 - 49	3.687	(2.790 - 4.871)*	4.057	(3.003 - 5.479)*
	50 - 59	2.364	(1.958 - 2.854)*	3.221	(2.572 - 4.033)*
	60 - 69	2.056	(1.791 - 2.359)*	2.298	(1.938 - 2.724)*
	70 - 80	1.643	(1.437 - 1.878)*	1.889	(1.589 - 2.233)*
	80 - 90	1.330	(1.139 - 1.553)*	1.661	(1.402 - 1.967)*
	>90 yr old	1.632	(1.213 - 2.196)*	1.446	(1.123 - 1.863)*

HR: hazard ratio; 95%CI: 95% confidence intervals for HR; yr: years. ^a^ 95%CIs that do not include the value 1 are marked by an asterisk.

The most significant limitation of this study was its reliance on self-reported data on the presence of comorbidities (information bias). The frequency of people who classified themselves as obese can be lower than the actual proportion of obese individuals^9^, and this prevents us from knowing the actual impact of obesity on COVID-19 fatality in this population. In this dataset, classes of pulmonary and cardiac comorbidities were not specified (for example, information regarding asthma, pulmonary fibrosis, sarcoidosis, arterial hypertension, and other conditions of interest were not available). The second limitation is the use of a secondary database, which may be subject to underreporting. Another important limitation concerns the definition of the time-to-event variable. Survival analysis was based on the time from diagnosis to death, though the time from infection to diagnosis can vary greatly from one individual to another. We believe that these data are not subject to lead-time bias, given that all diagnoses were probably made in the clinical phase of the disease, after the onset of symptoms, due to the absence of effective screening programs during the course of the pandemic. However, the maximum incubation period is assumed to be up to 14 days, and the search for medical care does not always occur at the onset of symptoms, which contributes to the time from diagnosis to death being a potentially biased variable to characterize the time to death of an infected individual.

Despite these shortcomings, the results from this study show that COVID-19-positive individuals, presenting with conditions such as obesity, diabetes, and renal, pulmonary, and cardiovascular comorbidities, are at a higher fatality risk than individuals without these chronic diseases, which is in agreement with previous reports. For example, a systematic review and meta-analysis including 17 articles with a total of 543,399 patients also showed that chronic kidney disease, diabetes, and obesity are associated with an increased risk of death from COVID-19^10^. In addition, a cross-sectional study including 889 people hospitalized due to COVID-19 in ES showed higher mortality among those with comorbidities and users of public hospitals^11^. In a study that included 2,070 confirmed cases of the disease reported in Ceará, a Brazilian state located in the Northeast region, the highest risk of COVID-19 death was observed in people with cardiovascular disease, neurologic disease, and pneumopathies^12^. Gacche et al.^13^ reported a comprehensive review of the relationship between mortality of COVID-19 patients and diabetes, obesity, and other chronic conditions, with special attention to the pathophysiology of the disease.

Our results suggest that elevated HRs associated with comorbidities are more marked in the younger age groups and, as reported by Ge et al.^14^, the young population with comorbidities should also be considered as a vulnerable group. In addition, our results suggest a strong association between the level of education and fatality risk due to COVID-19, showing that strategies for the prevention and management of the disease also need to consider the social aspects of the population. As reported by Horton in a syndemic perspective^15^, the search for a solution to the COVID-19 crisis should not be purely biomedical, but more attention to non-communicable diseases and socioeconomic inequality is required.

It should be noted that the present study was made possible by the availability of non-aggregated data from the COVID-19 Panel^4^. Unfortunately, COVID-19 data are mostly available to the public as summary or aggregate count files, in a format not suitable for statistical analyses, such as those presented in this paper.
